# Interleukin-6 Signaling in Triple Negative Breast Cancer Cells Elicits the Annexin A1/Formyl Peptide Receptor 1 Axis and Affects the Tumor Microenvironment

**DOI:** 10.3390/cells11101705

**Published:** 2022-05-20

**Authors:** Lara Vecchi, Sara Teixeira Soares Mota, Mariana Alves Pereira Zóia, Isabella Castro Martins, Jessica Brito de Souza, Tiago Góss Santos, Adriano de Oliveira Beserra, Victor Piana de Andrade, Luiz Ricardo Goulart, Thaise Gonçalves Araújo

**Affiliations:** 1 Laboratory of Nanobiotechnology, Institute of Genetics and Biochemistry, Federal University of Uberlândia, Campus Umuarama, Bloco 2E, Sala 248, 38400-902 Uberlândia, Brazil; saratsm.s@hotmail.com (S.T.S.M.); marianazoia@hotmail.com (M.A.P.Z.); isabellacastroisabella@gmail.com (I.C.M.); souzajessicab34@gmail.com (J.B.d.S.); 2 Laboratory of Genetics and Biotechnology, Federal University of Uberlândia, 38413-163 Patos de Minas, Brazil; 3 International Research Center, AC Camargo Cancer Center, 01509-900 São Paulo, Brazil; tsantos@accamargo.org.br (T.G.S.); profadrianoliveira@hotmail.com (A.d.O.B.); 4 Department of Investigative Pathology, AC Camargo Cancer Center, 01525-001 São Paulo, Brazil; victorpiana@me.com

**Keywords:** Annexin A1, autocrine signaling, breast cancer, formyl peptide receptor, IL-6, STAT3

## Abstract

Annexin A1 (AnxA1) is a pleiotropic protein that exerts essential roles in breast cancer (BC) growth and aggressiveness. In our previous work, we described the autocrine signaling of AnxA1 through formyl peptide receptor 1 (FPR1) in the triple-negative (TN) BC cell line, MDA-MB-231. Here, we aimed to describe the interaction between the AnxA1/FPR1 and the Interleukin-6 (IL-6) signaling pathways and their role in the tumor microenvironment (TME). First, we demonstrated that AnxA1 and IL-6 expression levels are correlated in BC tissue samples. In three TNBC cell lines, overexpression of both AnxA1 and IL-6 was also identified. Next, we inhibited FPR1, the IL-6 receptor and STAT3 in both MDA-MB-231 and MDA-MB-157 cells. The FPR1 inhibition led to increased levels of IL-6 and secreted AnxA1 in both cell lines. On the other side, inhibition of the IL-6 receptor or STAT3 led to the impairment of AnxA1 secretion, suggesting the essential role of the IL-6 signaling cascade in the activation of the AnxA1/FPR1 autocrine axis. Finally, we described the interaction between IL-6 and the AnxA1/FPR1 pathways and their role on the TME by analyzing the effect of supernatants derived from MDA-MB-231 and MDA-MB-157 cells under the inhibition of FPR1 or IL-6 signaling on fibroblast cell motility.

## 1. Introduction

Breast cancer (BC) is the most common cancer worldwide and is a major cause of morbidity and mortality in women [1,2]. The expression of estrogen receptor (ER) and progesterone receptor (PR)—that are collectively named hormone receptors (HR)—human epidermal growth factor receptor 2 (HER2) and of the proliferation marker, Ki67, defines BC subtypes which are categorized into: Lumina A (HR+, HER2-), Luminal B (HR+, HER2+ or HR+, HER2-; Ki67 high), HER2-enriched (HR-, HER2+), and Triple-Negative (TN: HR-, HER2-). The majority of TNBCs belongs to the basal-like subtype and displays a highly aggressive phenotype with poorer overall survival and high relapse rates [3,4]. Although TNBC can show an initial good response to chemotherapies, it frequently develops resistance and, due to the limitations of available targeted therapies, it still represents a deadly disease [5,6].

The association between inflammation and cancer is unquestionable [7] since chronic inflammation frequently predisposes cells to oncogenic transformation [8]. A key mediator of the anti-inflammatory response is the 37 kDa phospholipid-binding protein Annexin A1 (AnxA1). In addition to regulating membrane trafficking, proliferation, differentiation, and apoptosis [9,10,11,12], AnxA1 inhibits Phospholipase A2 (PLA2) in the cytoplasm [13,14] and impedes the release of arachidonic acid, which is a key contributor to the development of the tumor microenvironment (TME). AnxA1 also elicits, upon its secretion, the formyl peptide receptor (FPR) signaling pathway that is responsible for inhibiting leukocyte adhesion and migration [15,16]. Therefore, AnxA1 is a pleiotropic protein with effects beyond the immune system and is implicated in the promotion and progression of different diseases [17].

In the last decade, researchers have focused on the correlation between AnxA1 and the aggressiveness of different tumors [18,19,20,21]. In BC context, higher expression levels of AnxA1 have been described for the TNBC subtype [22,23] and the role of this protein in inducing the epithelial-mesenchymal transition (EMT) in BC has also been depicted [24]. AnxA1 can be found in cytoplasm, nuclei and supernatants of the TNBC cell line, MDA-MB231, in its 37 kDa intact form and in its cleaved form of 33 kDa [23]. Notably, findings showed that the intact form displays anti-inflammatory activity while its cleaved form seems to be pro-inflammatory [25]. Interestingly, previous works showed an association between AnxA1 and the inflammatory cytokine, IL-6. On one side, it has been demonstrated that IL-6 stimulates the upregulation and secretion of AnxA1 by acting on an IL-6 responsive region within the AnxA1 promoter [26]. On the other side, it has been demonstrated that AnxA1 signaling through FPR2 inhibits the IL-6 expression in neutrophils [27] and that AnxA1 signaling through FPR1 inhibits the IL-6 expression in the TNBC cell line MDA-MB-231 [23].

IL-6 is one of the main mediators of the inflammatory response [28] with an important role in host immune defense mechanisms and in the modulation of cellular growth [29]. IL-6 signals through a cell-surface receptor, the IL-6 receptor (IL-6R), and through a soluble form of IL-6 receptor (sIL-6R) [30], leading to the activation of the transcription factor STAT3 [31]. IL-6 modulates the proliferation and differentiation of malignant cells [32] and its expression is frequently increased in different tumors including prostate cancer [33] and BC [34]. In BC, the overexpression of both IL-6 and IL-6 receptors (IL-6R and sIL-6R) was observed [35] and high serum levels of IL-6 have been correlated with a poorer prognosis [36] and metastasis to bones [37]. Moreover, the IL-6 expression levels are significantly higher in the basal-like BC phenotype [36] and in multi drug-resistant cancer cell lines [38,39]. Similar findings were observed for STAT3 whose overexpression levels correlate with poorer survival rates in patients with solid tumors [40] and with the resistance of cancer cells towards chemotherapy [41]. In BC, STAT3 stimulates proliferation, survival, angiogenesis, chemoresistance and promotes EMT and metastasis [31,42].

In this work, we describe the functional interaction between AnxA1 and IL-6 in BC and unravel how this interaction could maintain the malignant phenotype of TNBCs. By using tissue microarrays (TMAs) and BC cell lines, we observed that AnxA1 expression correlates with the expression of IL-6. TNBC cells are characterized for high levels of both AnxA1 and IL-6 and for the activation of an autocrine signaling by AnxA1. We found that IL-6 increased AnxA1 externalization and, in this way, supported the AnxA1 autocrine signaling cascade through the activation of FPR1 in TNBC cell lines. When FPR1 was inhibited in TNBC cells, the expression levels of IL-6 were increased as an attempt to sustain the autocrine signaling of AnxA1. We also demonstrated that the monoclonal antibody against IL-6 receptor, Tocilizumab (TCZ), inhibited the in vivo growth of MDA-MB-231 tumors in mice, reducing metastasis formation. Lastly, supernatants derived from MDA-MB-231 and MDA-MB-157 cells previously treated with inhibitors of FPR1 and/or IL-6 influenced the motility of fibroblast cells. Taken together, our data suggest that in aggressive BCs, the AnxA1 and IL-6 signaling pathway cascades are linked by a biunivocal compensatory relation.

## 2. Materials and Methods

### 2.1. Cell Culture and Treatments

The non-tumorigenic human breast cell line, MCF-10A, and the human BC cell lines, MCF-7 (ER+, PR+), MDA-MB-453 (HER2+), MDA-MB-231, MDA-MB-157, and BT-459 (TNBC cells) were obtained from the American Type Culture Collection (ATCC). All the BC cell lines were cultured in IMDM (Thermo Fisher Scientific, Dallas, TX, USA) while MCF-10A cells were cultured in DMEM F12 (Thermo Fisher Scientific) supplemented with EGF (20 ng/mL), hydrocortisone (0.5 µg/mL), cholera toxin (100 ng/mL), and insulin (10 µg/mL) (Thermo Fisher Scientific). Fibroblast cells (HFF) were cultured in RPMI (Thermo Fisher Scientific). All media were supplemented with 10% of Fetal Bovine Serum (Thermo Fisher Scientific) and 1× antibiotic-Antimycotic (Thermo Fisher Scientific). All cells were free from mycoplasma and were authenticated through STR analysis.

Cells were treated with the following chemicals and antibodies diluted in serum-free medium: FPR antagonist Cyclosporin H (CsH-Sigma, used at 1 µM), neutralizing anti-IL-6 antibody (Tocilizumab Actemra, Roche, used at 2 µg/mL), STATTIC (STC-Sigma 1 and 5 µM) and recombinant IL-6 (rIL-6, Thermo Fisher Scientific, used at 1 µg/mL).

### 2.2. Cell Lysis and Immunoblotting

Nuclear and cytoplasmic protein extracts of 24 h-treated cells were prepared by using NE-PER Nuclear and Cytoplasmic Extraction Reagent kit (Thermo Fisher Scientific) following the manufacturer’s instructions. Protein concentration was measured using the BCA assay (Pierce™ BCA™ ProteinAssay; Thermo Fisher Scientific). Identical amounts of protein extracts and supernatants were separated electrophoretically on 10% SDS-PAGE and transferred onto a nitrocellulose membrane (Hybond, GE Healthcare, Chicago, IL, USA). Membranes were blocked with PBS 5% milk for 1 h and subsequently incubated with anti-AnxA1 (1:2000; 71-3400; Thermo Fisher Scientific). Anti-β-Actin (1:1000, SC-130656; Santa Cruz Biotechnology, Dallas, TX, USA) and anti-Lamin B2 (1:1000, SAB2702205, Sigma) were used as loading controls of cytoplasmic and nuclear extract, respectively. As a secondary antibody, the anti-rabbit HRP-conjugated (1:3000; G21234; Thermo Fisher Scientific, Carlsbad, CA, USA) was used.

### 2.3. Flow Cytometry

To analyze the activation of ERK1/2 and STAT3 and the expression levels of FPR1 and FPR2, cells were fixed and permeabilized by BD Cytofix/Cytoperm™ Fixation/Permeabilization Solution Kit (BD Pharmingen, San Jose, CA, USA). Subsequently, cells were stained with anti-ERK1 (1:100; (phospho T202/Y204) + ERK2 (phospho T185/Y187); ab32538, Abcam)); anti-STAT3 (1:100; phospho Y705 AF4607; R&D Systems, Minneapolis, MN, USA); anti-FPR1 FITC-conjugated (1:50; FAB3744F; R&D Systems); and anti-FPR2 PE-conjugated (1:50; FAB3479P; R&D Systems). Anti-rabbit IgG-FITC (1:200, 656111, Thermo Fisher Scientific) was used as a secondary antibody for ERK1/2 and STAT3 staining. The isotype controls for FPR1 ((anti-mouse IgG2A-FITC control) 1:50; IC003F; R&D Systems) and FPR2 ((anti-mouse IgG2B-PE control) 1:50; IC0041P, R&D Systems) were also used.

### 2.4. Calcium Flux

In order to assess the intracellular calcium increase, cells were treated for 24 h with STC, TCZ, CsH, or rIL-6. The next day, cells were treated for an additional hour, washed with Hanks’ Balanced Salt Solution (HBSS), and incubated with a mixture of the Fluo4 AM calcium dye and Pluronic acid (Thermo Fisher Scientific) for 30 min at room temperature (RT) in the dark. Cells were washed twice with HBSS to remove the unloaded dye. A standard curve of calcium was prepared in HBSS buffer without calcium. The cytosolic calcium in samples was measured by using a fluorescence microplate reader (Molecular Devices, Menlo Park, CA, USA) using excitation.

### 2.5. Immunohistochemistry

Tissue microarray (TMA) slides containing samples of 1 mm diameter and 1 µM thickness of BC primary tumors (US Biomax BR1503d) were used. All samples were clinicopathologically characterized according to Scarff Bloom-Richardson grading, TNM staging system and ER, PR and HER2 status.

TMA slides were incubated for 16 h with anti-IL-6 (Thermoscientific AHC0762) or anti-AnxA1 (71-3400; Thermoscientific) at 1:20 and 1:500 dilutions, respectively, and subsequently incubated with Novolink™ Max Polymer Detection System (Leica Biosystems, Newcastle, UK) with HRP polymer (HRP Multimer). Next, the reactions were developed with diaminobenzidine substrate solution (DAB, Sigma). The slides were counterstained with Harris hematoxylin (Dinamica Quimica Comtemporanea, Indaiatuba, SP, Brazil), mounted on Tissue-Tek film (Sakura), and digitalized by using the Aperio ScanScope XT digital scanner (Leyca Biosystems, CA, USA). Analyses were performed using the PixelCount V9.0 algorithm.

### 2.6. In Vivo Assay

The Ethics Committee of the Institutional Research Board of the AC Camargo Cancer Center approved all procedures involving mice, under the number 072/2015. All experiments were performed in accordance with the relevant guidelines and regulations.

Female athymic nude mice (2–4 months old) were subcutaneously inoculated with 1 × 10^6^ MDA-MB-231-Luc cells. After tumors reached 300 mm^3^, animals were randomized into two groups. The treatments were carried out intraperitoneally with PBS + DMSO 5% (group control, *n* = 11) or with TCZ 10 mg/kg (*n* = 7) three times a week for 1 month or if tumors reached 1000 mm^3^. After treatments, tumors were resected, and metastases were monitored in vivo by whole animal imaging with bioluminescence (IN-VIVO FX Pro, Bruker, Billerica, MA, USA).

### 2.7. Wound-Healing Assay

Cell migration was measured by in vitro wound-healing assay. Briefly, by using a pipette tip, wounds on HFF cell monolayers were created. Subsequently, cells were washed twice with PBS (phosphate-buffered saline) and treated with mitomycin C (Sigma, used at 10 µg/mL) to inhibit mitosis. Afterwards, cells were treated with supernatants of MDA-MB-231 or MDA-MB-157 cells not treated or treated with 1 µM CsH, 2 µg/mL of TCZ, 5 µM STC and 1 µg/mL of rIL-6. Pictures of the wounds (10×) were taken at times 0 and 24 h (EVOS, AMG). By comparing the gap at times 0 and 24 h, the percentages of gap closure were obtained.

### 2.8. ELISA

IL-6 expression was analyzed by ELISA assay (Thermo Fisher Scientific, Waltham, MA, USA) in supernatants from the human BC cell lines. TNBC cell lines, MDA-MB-231 and MDA-MB-157 were treated or not for 24 h with STC, TCZ, CsH or rIL-6 in serum-free medium.

### 2.9. Quantitative Real-Time PCR

Total RNAs from the breast cell lines were extracted using Trizol Reagent (Thermo Fisher Scientific, Waltham, MA, USA) according to the manufacturer’s recommendations. The RNA concentration and quality were analyzed spectrophotometrically by absorbance readings at 260 and 280 nm, and 1 μg was used to synthesize cDNA using GoScript Reverse Transcription Mix, Oligo(dT) (Promega; Madison, WI, USA), following the supplier’s instructions.

The real-time PCR (qPCR) was conducted in an ABI PRISM 7300 Sequence Detection System (Applied Biosystems, Carlsbad, CA, USA) with 5.0 μL of Power SYBR Green PCR Master Mix (Applied Biosystems) and 0.5 μM of primers designed for *ANXA1* (5′-GATTCAGATGCCAGGGCCT-3′ and 5′-ATCCACAACTTCGCAGAGTG-3′), *IL-6* (5′-AACCACGGCCTTCCCTACTT-3′ and 5′-GAGTTGTGCAATGGCAATTCTG-3′) and *STAT3* (5′-CCAGTTTACCACGAAAGTCAGG-3′ and 5′-AAAGACTCTGGGGATGTTGCTG-3′). Beta-2-microglobulin (*B2M*) gene was used as reference for relative quantification and the primers were published previously [19]. Cycling conditions were as follows: 95 °C for 10 min, followed by 40 cycles of 95 °C for 15 s and 60 °C for 60 s. Each sample was tested in triplicate and mRNA relative levels were calculated using the ΔΔCq method. *β2M* mRNA levels did not differ across samples.

### 2.10. AnxA1 Knockdown

Lentiviral particles encoding a scrambled shRNA sequence (1.0 × 10^5^ IFU; shControl, sc-108080, Santa Cruz Biotechnology) or a pool of lentiviral particles encoding four shRNAs specific for AnxA1 knockdown (1.0 × 10^5^ IFU; shAnxA1, sc-29198, Santa Cruz Biotechnology) were transduced in MDA-MB-231 cells through a previous incubation with Polybrene at 5 μg/mL (Santa Cruz Biotechnology) followed by infection with the virus particles. Clones of cells expressing the shAnxA1 were selected by using 5 μg/mL of puromycin (Santa Cruz Biotechnology).

### 2.11. Statistical Analysis

The Student’s *t* test was used to analyze significant differences between means. Correlations were calculated by Pearson’s coefficient, McNemar test and Odds ratios (OR). OR with 95% confidence intervals (CI) were estimated using unconditional logistic regression. Statistical significance was considered when *p* < 0.05. The statistical analyses were performed by using GraphPad Prism 7.0 (GraphPad Software Inc., La Jolla, CA, USA) and SPSS version 17.0 (SPSS; Chicago, IL, USA).

## 3. Results

### 3.1. Correlation between AnxA1 and IL-6 Expression in BC Samples

A total of 106 patients were included in this study. The patients’ average age was 50.8 years, ranging from 28 to 80 years. ER was expressed in 41 (38.7%) patients, PR was expressed in 28 patients (26.4%), and HER2 was expressed in 39 patients (36.8%). In addition, breast tumors were classified according to their molecular subtypes: 37 Luminal A (35%), 11 Luminal B (10.4%), 26 HER2-enriched (24.5%); and 32 patients were defined as TNBC (30.1%). No axillary lymph node involvement was observed in 37 patients (34.9%), while in 69 patients, one or two (N1-2) lymph node metastases were identified (65.1%). The distribution based on tumor size was as follows: T1, 6 (5.7%); T2, 73 (68.9%); T3, 19 (17.9%) and T4, 8 (7.5%). The distribution by stage was stage I, 3 patients (2.8%); stage II, 73 (68.9%); and stage III, 30 (28.3%). For histological grading of tumors, 11 samples (10.4%) were GI; 82 (77.3%) GII, and 13 (12.3%) GIII.

**Figure 1:**
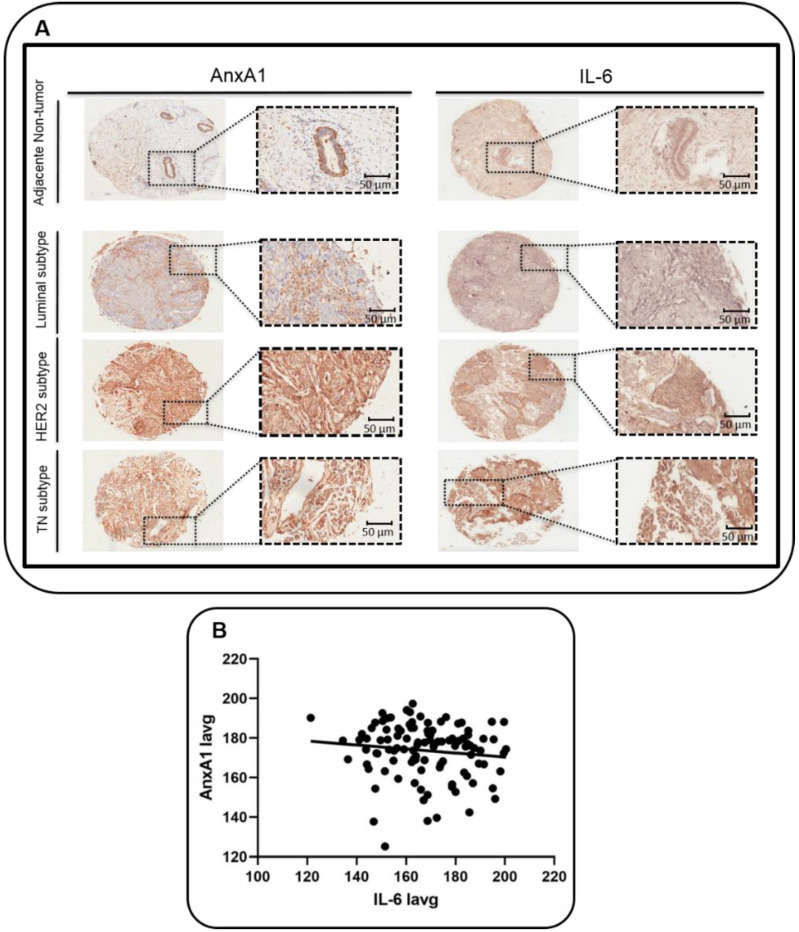
**Expression of Annexin A1 (AnxA1) and Interleukin-6 (IL-6) in tumor and adjacent non-tumor breast tissues.** (**A**) Representative images of adjacent non-tumor breast tissue, and of the tumor subtypes Luminal, HER2-enriched and Triple negative (TN). Tissue microarrays (TMA) were stained for AnxA1 and IL-6 through immunohistochemistry. Outset shows the magnified image of the corresponding panel (20 × with the outset showing a 40 × aperture magnified view of the same TMA spot). Scale bar = 50 μm (magnification). (**B**) Correlation between the expression of AnxA1 and IL-6 evaluated by linear regression (R^2^ = 0.96 and *p* < 0.00001). Iavg: average intensity of all pixels.

Samples have been analyzed for the expression of AnxA1 and IL-6 and as a control of the staining a TNBC sample has been incubated with all reagents but no primary antibody (Figure S1). We found that in adjacent non-tumor breast tissues, both AnxA1 and IL-6 were expressed in glandular and in the myoepithelial cell layer. AnxA1 and IL-6 were weakly expressed in the Luminal subtype, while moderately expressed in the HER2-enriched subtype and highly expressed in the TNBC subtype (Figure 1A). We also found a positive correlation using the Person’s test and linear regression between AnxA1 and IL-6 (Figure 1B; R = 0.98 and R^2^ = 0.96 and *p* > 0.00001).

In order to differentiate the expression of AnxA1 and IL-6 proteins according to clinicopathological data, samples were categorized into two groups (high and low) and analyzed using the Odds ratio test. With the aid of the pathologist’s visual score, we chose Iavg (Iavg: average intensity of all pixels) cut-off of 166.7 for IL-6 and 175.0 for AnxA1. The AnxA1 expression was 3.3 higher in TNBC when compared to other subtypes (Table 1; OR = 3.3; *p* = 0.007). Already, the IL-6 expression was lower in samples classified as N1 and N2 (Table 1; OR = 0.3; *p* = 0.003) and IL-6 was 2.6-fold less expressed in BC samples when they were not classified as Luminal A (Table 1; OR = 2.6; *p* = 0.03).

To conclude, it was possible to infer that the AnxA1 expression positively correlates with the IL-6 expression (R^2^ = 0.96 and *p* < 0.00001). Moreover, the AnxA1 and IL-6 expression negatively correlated with the Luminal A subtype, and the AnxA1 expression was higher in the TN subtype (Table 1).

### 3.2. TNBC Cell Lines Express High Levels of AnxA1 and IL-6

**Figure 2:**
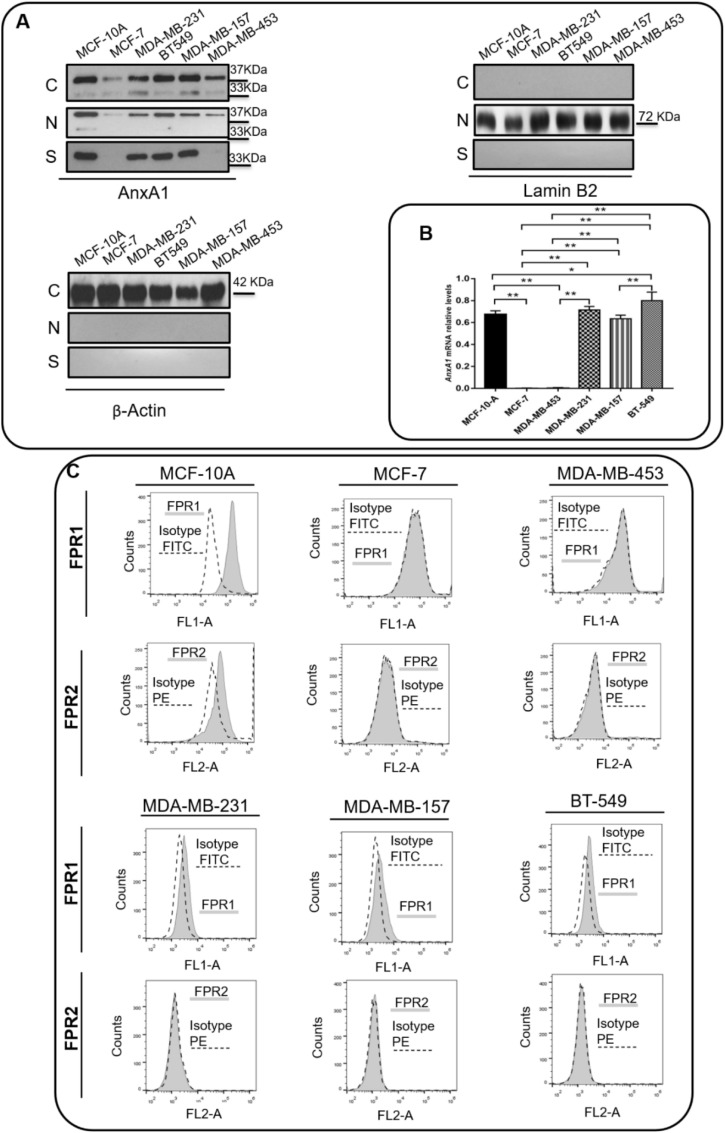
**TNBC cell lines express Annexin A1 (AnxA1).** (**A**) The cropped Western blotting of AnxA1 expression in cytoplasm (C), nuclei (N), and supernatants (S) of MCF-10, MCF-7, MDA-MB-231, BT-549, MDA-MB-157 and MDA-MB-453 cells. Full length AnxA1 (37 kDa) and the cleaved AnxA1 form (33 kDa) were detected. β-Actin (42 kDa) and Laminin B2 (72 kDa) were used to check the proper cell fractionation and as loading controls of cytoplasmic and nuclear extracts, respectively. (**B**) *ANXA1* mRNA relative level of MCF-10, MCF-7, MDA-MB-231, BT-549, MDA-MB-157 and MDA-MB-453 cells. (**C**) Expression analysis of AnxA1 receptors (FPR1 and FPR2) on the cell surface of MCF-10, MCF-7, MDA-MB-231, BT-549, MDA-MB-157 and MDA-MB-453 cells by flow cytometry (black peak with tracing line). As the negative control, the secondary antibody alone was used (solid gray peak). Three independent experiments were performed. * *p* < 0.05; ** *p* < 0.001.

The expression of AnxA1 and its receptors, FPR1 and FPR2, was assessed in a non-tumorigenic breast cell line (MCF-10A), in ER-positive BC cell line (MCF-7), in HER2-positive cell line (MDA-MB-453), and TNBC cell lines (MDA-MB-231, MDA-MB-157, and BT-459). As previously described [23], the AnxA1 protein is expressed in the cytoplasm, nuclei and supernatants of MCF-10A cells. Lower levels of AnxA1 were detected in the cytoplasm and nuclei of MCF-7 and MDA-MB-453 cells and were undetectable in supernatants of these lineages. The TNBC cell lines MDA-MB-231, MDA-MB-157 and BT-459 displayed an intense expression of AnxA1 in the cytoplasm, nuclei, and supernatants (Figure 2A,B). Either in the cytoplasm or nuclei, it was possible to visualize AnxA1 in its intact form (37 kDa) and in its cleaved form (33 kDa). In the supernatants of TNBC cells, AnxA1 was abundantly expressed in its cleaved form. Figure S2 shows a whole membrane staining to demonstrate the specificity of antibodies and proper cell fractionation. Regarding FPRs expression, MCF-7 and MDA-MB-453 cells did not express either FPR1 or FPR2; MCF-10A cells expressed both FPR1 and FPR2, whereas MDA-MB-231, MDA-MB-157 and BT-459 cells expressed FPR1 only (Figure 2C).

**Figure 3:**
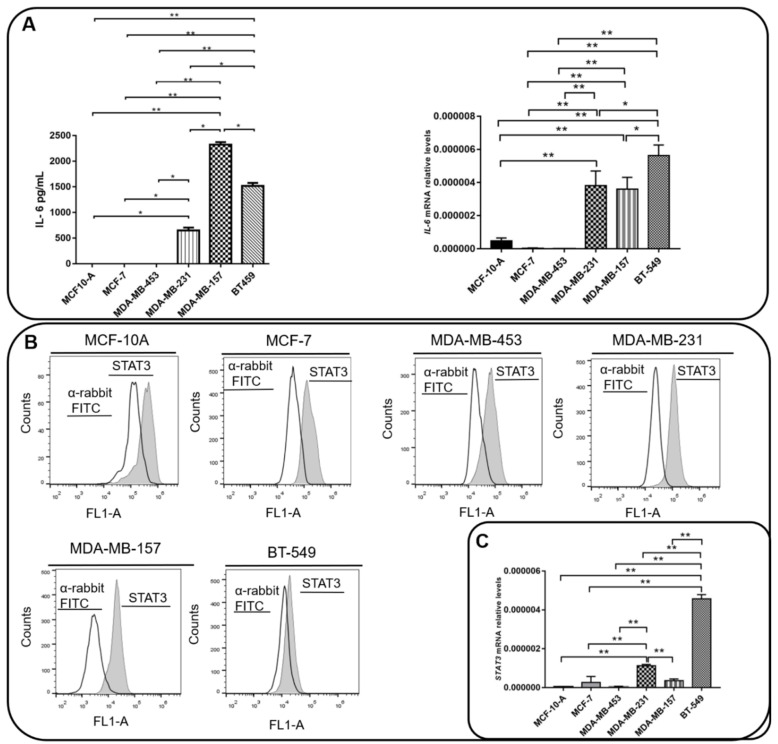

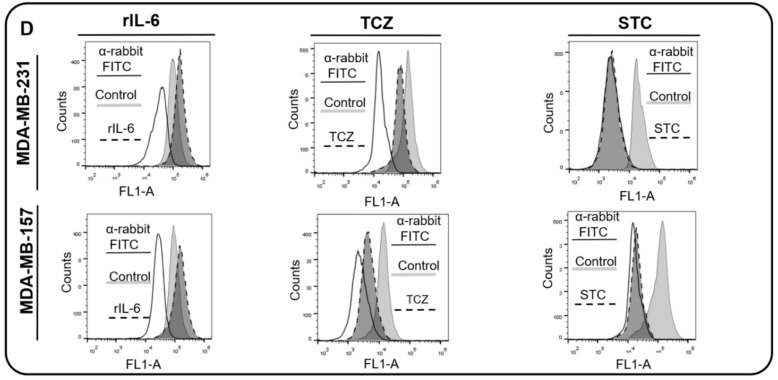
**Interleukin 6 (IL-6) and STAT3 expression in BC cells.** (**A**) IL-6 levels were measured by ELISA assay in supernatants of MCF-10, MCF-7, MDA-MB-231, BT-549, MDA-MB-157 and MDA-MB-453 cells. *IL-6* mRNA relative levels of MCF-10, MCF-7, MDA-MB-231, BT-549, MDA-MB-157 and MDA-MB-453 cells were also recorded. (**B**) Expression of activated STAT3 (STAT3^pY705^) in MCF-10, MCF-7, MDA-MB-231, BT-549, MDA-MB-157 and MDA-MB-453 cells was analyzed by flow cytometry (gray peak). As the negative control, the secondary antibody alone was used (white peak). (**C**) *STAT3* mRNA relative levels of MCF-10, MCF-7, MDA-MB-231, BT-549, MDA-MB-157 and MDA-MB-453 cells. (**D**) STAT3 phosphorylation levels (STAT3^pY705^-dark gray peak with tracing line) were analyzed in MDA-MB-231 and MDA-MB-157 cells treated with Tocilizumab (TCZ), recombinant IL-6 (rIL-6) and STAT3 inhibitor, STATTIC (STC). As the negative control, the secondary antibody alone was used (white peak). * *p* < 0.05; ** *p* < 0.001. Three independent experiments were performed.

We next analyzed the expression of IL-6 and the activation of STAT3 in the BC cell lines used in this study. We found that MDA-MB-231, MDA-MB-157, and BT459 cells secrete high amounts of IL-6 in the extracellular milieu, while IL-6 expression levels in MCF10A, MCF-7, and MDA-MB-453 cells were undetectable (Figure 3A). The levels of activation of STAT3 (STAT3^pY705^) were similar among all cell lines (Figure 3B,C), except for BT-549, which expressed lower levels of STAT3^pY705^. In order to assess whether the IL-6 expression was responsible for the activation of the STAT3 pathway in TNBC cells, we inhibited or activated the IL-6 signaling in MDA-MB-231 and MDA-MB-157 cells, by using TCZ or a rIL-6, respectively. As a control, we used cells treated with the vehicle only and cells treated with the inhibitor of STAT3, STC. As expected, STC inhibited the activation of STAT3 as visualized by the decrease in STAT3^pY705^ levels. TCZ significantly impaired STAT3 phosphorylation, while in cells treated with rIL-6, an increase in STAT3 activation levels was observed (Figure 3D).

Taken together, these results showed that there is a concordance in the expression of AnxA1 and IL-6 in the supernatant of all the BC cell lines studied. Moreover, higher levels of both molecules were observed in the cytoplasm, nuclei, and supernatant of TNBC cells. On the contrary, STAT3 activation levels are similar among cell lines, except for BT-549. We, therefore, showed that the IL-6 signaling pathway might be of the mechanisms that activate STAT3 in MDA-MB-231 and MDA-MB-157 TNBC cells.

### 3.3. AnxA1 Autocrine Signaling Leads to a Decrease in IL-6 Expression

**Figure 4:**
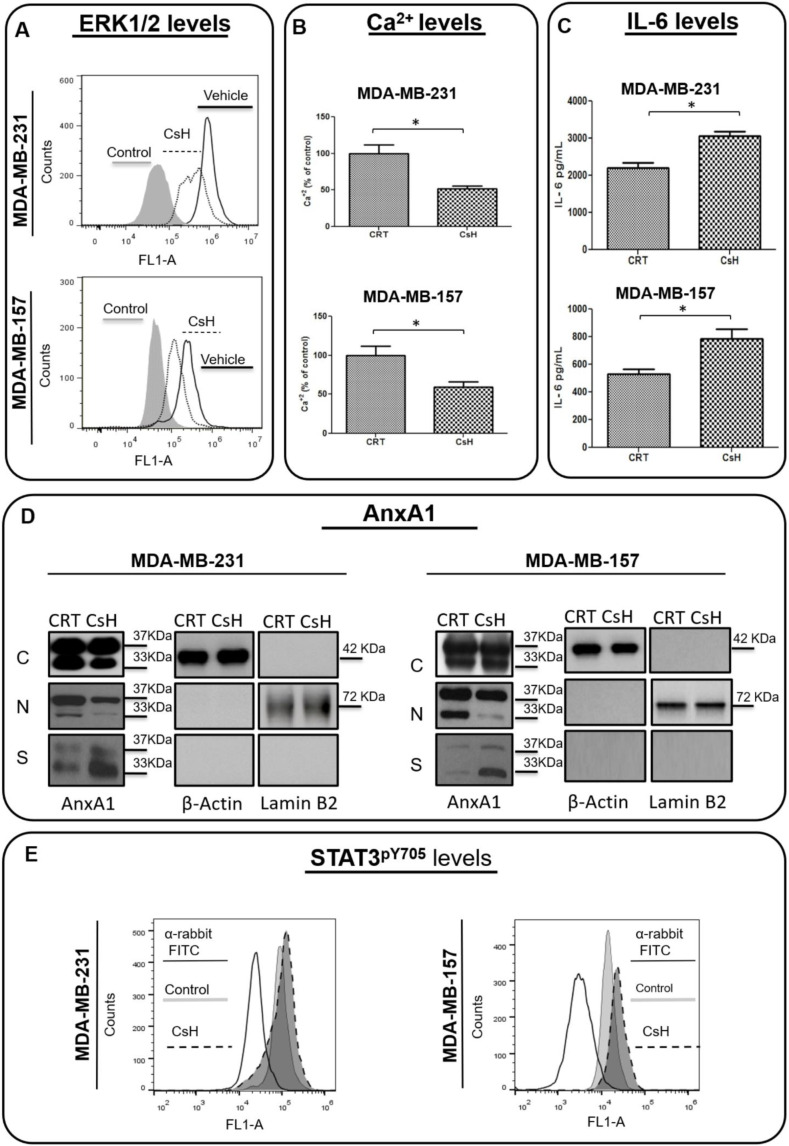
**Effects of Cyclosporin H (CsH) treatment on the Annexin A1 (AnxA1) expression and formyl peptide receptor 1 (FPR1)-downstream cellular events in MDA-MB-231 and MDA-MB-157 cells.** All treatments were conducted with 1 μM of CsH for 24 h. (**A**) The expression of phosphorylated ERK1/2 (ERK1^pT202/Y204^ + ERK2^pT185/Y187^) was measured using flow cytometry in treated and not-treated control cells. (**B**) Cytosolic calcium levels were measured by using Fluo 4 AM in MDA-MB-231 and MDA-MB-157 cells in the presence or absence of CsH. (**C**) IL-6 levels were measured by the ELISA assay in the supernatant of cells. (**D**) The cropped Western blotting of the AnxA1 expression in the cytoplasm (C), nuclei (N), and supernatants (S) of MDA-MB-231 and MDA-MB-157 cells treated or not treated (control) with CsH. β-Actin and Lamin B2 were used to check the proper cell fractionation and as loading controls of cytoplasmic and nuclear extracts, respectively. (**E**) STAT3 phosphorylation levels (STAT3^pY705^) were analyzed in cells treated with CsH compared to control cells (not treated). * *p* < 0.05. Three independent experiments were performed.

As we previously described, the N-terminal peptide of AnxA1 induces an autocrine signaling in MDA-MB-231 cells [39]. In order to confirm the AnxA1 autocrine signaling in MDA-MB-157 cells, this lineage was treated with CsH and the effect on IL-6 expression, on ERK1/2 phosphorylation and on cytosolic calcium, and was compared with MDA-MB-231 cells. We found that ERK1/2 phosphorylation (Figure 4A), as well as cytosolic calcium concentration, were inhibited by CsH in MDA-MB-157 cells at a similar extent to what was observed in MDA-MB-231 cells (Figure 4B). Moreover, the inhibition of FPR1 by CsH increased IL-6 secretion in both cell lines (Figure 4C). We next compared, by Western blotting analysis, the effect of CsH on AnxA1 expression on the two TN cell lines. Again, the behavior was similar, since CsH slightly decreased the nuclear localization of AnxA1 while significantly increasing the secretion of AnxA1 in both cell lines (Figure 4D). As previously described [41], by knocking down AnxA1 in MDA-MB-231, it was possible to observe an increase in the secretion of IL-6. In accordance with the increased levels of IL-6 in the supernatant of cells treated with CsH, the levels of STAT3^pY705^ were also upregulated after treatment (Figure 4E).

**Figure 5:**
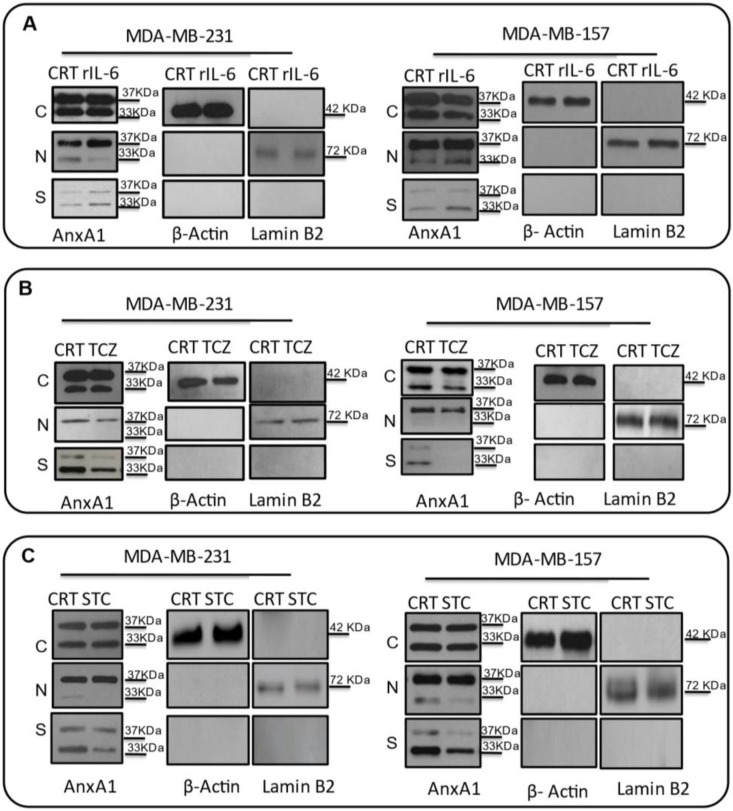
**Effects on the Annexin A1 (AnxA1) expression in MDA-MB-231 and MDA-MB-157 after the stimulation and inhibition of the IL-6 pathway.** The cropped Western blotting of AnxA1 expression in the cytoplasm (C), nuclei (N), and supernatants (S) of MDA-MB-231 and MDA-MB-157 cells. (**A**) Cells were treated for 24 h with 1 μM of recombinant human IL-6 (rIL-6). (**B**) Cells were treated for 24 h with 1 μM of neutralizing antibody anti-human IL-6 (Tocilizumab; TCZ), and (**C**) cells were treated for 24 h with 1 μM of an inhibitor of the IL-6 signaling pathway molecule, STAT3 (STATTIC; STC). β-Actin and Lamin B2 were used to check the proper cell fractionation and as loading controls of cytoplasmic and nuclear extracts, respectively. Three independent experiments were performed.

Based on these results, we further sought to test whether this increase in IL-6 could be, indeed, elicited in order to increase AnxA1 secretion and its signaling through FPR1. Then, we treated MDA-MB-231 and MDA-MB-157 cell lines with rIL-6, with a neutralizing antibody anti-human IL-6 (TCZ), and with an inhibitor of the IL-6 signaling pathway, STAT3 (STC). An increase in AnxA1 secretion was observed upon treating cells with rIL-6 (Figure 5A), while the treatment with either TCZ or STC inhibited AnxA1 secretion in both cell lines (Figure 5B,C). Taken together, these results suggest that the IL-6 signaling pathway is important in TNBC cells to stimulate the secretion of AnxA1.

**Figure 6:**
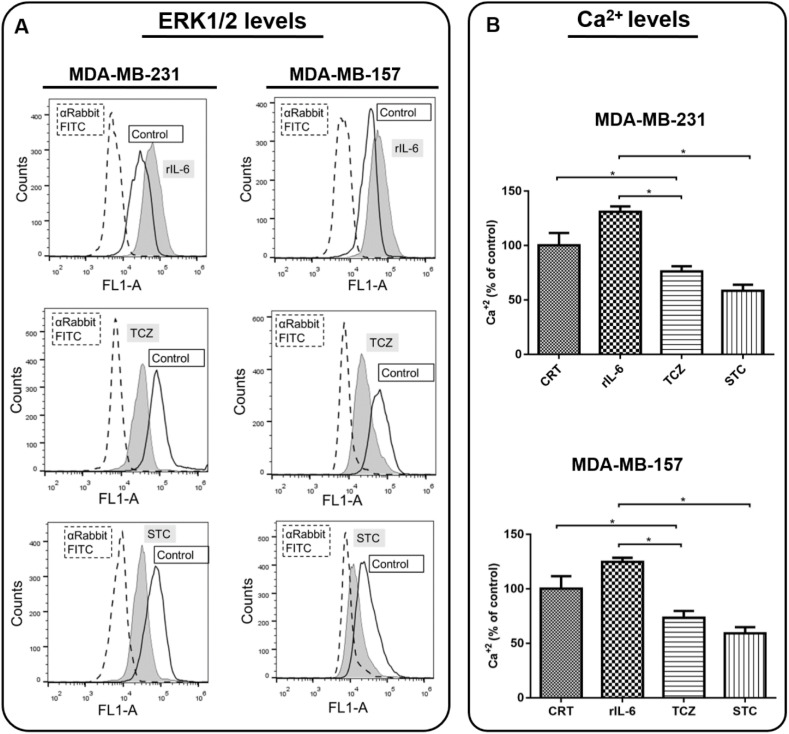
**Analysis of the Interleukin 6 (IL-6) signaling pathway downstream cellular events in MDA-MB-231 and MDA-MB-157**. Cells were treated with recombinant human IL-6 (rIL-6), with neutralizing antibody anti-human IL-6 (Tocilizumab; TCZ), and with an inhibitor of the IL-6 signaling pathway molecule, STAT3 (STATTIC; STC), for 24 h. Untreated cells were used as the control (**A**). The expression of phosphorylated ERK1/2 was measured using flow cytometry. (**B**) Cytosolic calcium levels were measured by using Fluo 4 AM. * *p* < 0.05. Three independent experiments were performed.

The activation of the ERK1/2 pathway and cytosolic calcium levels were also recorded. By analyzing the ERK1/2 phosphorylation levels by flow cytometry, we observed an increase in ERK1/2 phosphorylation upon treating cells with rIL-6 and, in contrast, we found that the treatment with either TCZ or STC was highly effective in inhibiting ERK1/2 activation (Figure 6A). Likewise, treatment with rIL-6 stimulated cytosolic calcium levels and either TCZ or STC decreased cytosolic calcium concentrations (Figure 6B).

These results demonstrated that IL-6 signaling is one of the mechanisms responsible for the activation of ERK1/2 and cytosolic calcium rise. We also observed that when the AnxA1 autocrine signaling is inhibited, there is an increase in the expression and autocrine signaling of IL-6. This raise in IL-6 represents a compensatory mechanism that TNBC cells activate in order to stimulate the AnxA1 autocrine signaling.

### 3.4. IL-6 Inhibition Is Involved in Tumor Growth

**Figure 7:**
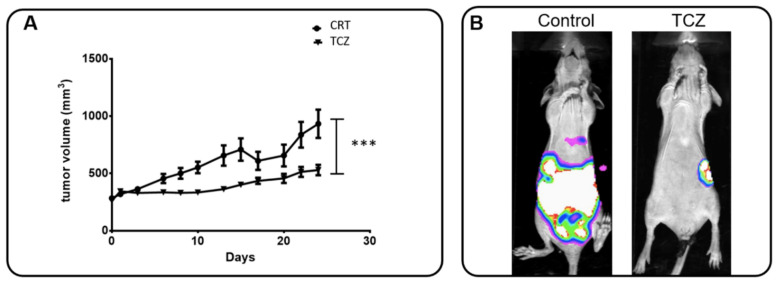

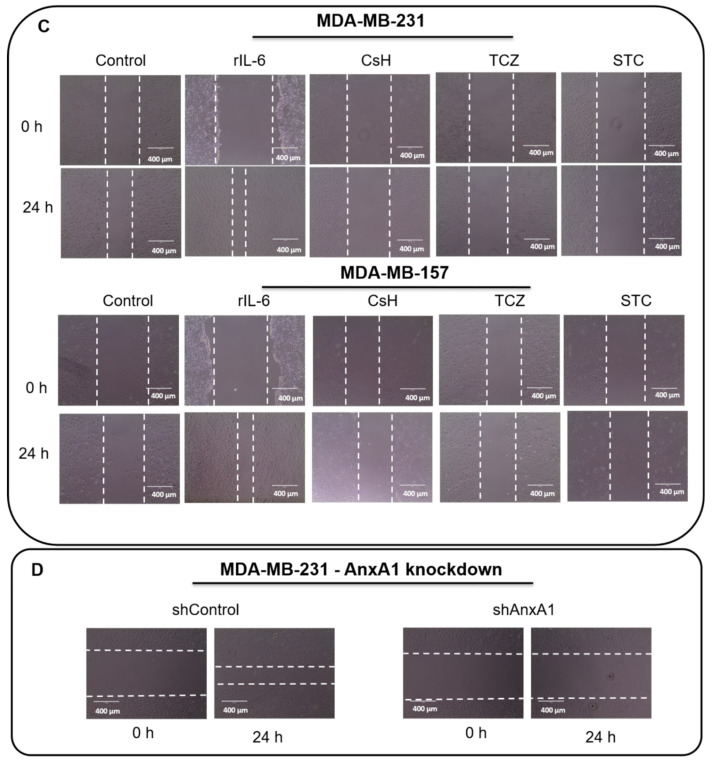
**Tocilizumab (TCZ) inhibits tumor growth and distal metastasis in the in vivo model and influences the motility of fibroblast cells.** (**A**) Female nude mice bearing MDA-MB-231 tumors were treated with the control solution (PBS + DMSO 5%) or TCZ (10 mg/kg) 3 times a week for 1 month. The volumes (in mm^3^) of primary tumors were measured at each corresponding time point. Date show that TCZ treatment totally inhibited (significantly at *p* = 0.0005, analyzed by Paired Student’s *t*-test) the primary tumor growth. (**B**) After tumor removal, tumor relapse and spontaneous metastasis occurrence were monitored via bioluminescence imaging and representative mice for groups were exemplified (control: *n* = 11; TCZ: *n* = 7). (**C**) The supernatants derived from MDA-MB-231 and MDA-MB-157 cells were used to evaluate the motility of fibroblast cells (HFF) by a wound-healing assay in the presence of recombinant human IL-6 (rIL-6), Cyclosporin H (CsH), STATTIC (STC) and TCZ. Cells were scratched with a cell scraper and photographed by phase-contrast 221 microscopy. Representative images show cell migration at 0 h and after 24 h. (**D**) The supernatants derived from the MDA-MB-231 cell line stably knocked down for AnxA1 (shAnxA1) were used to evaluate the motility of HFF and were compared to the MDA-MB-231 cell line stably expressing a control shRNA (shControl). Three independent experiments were performed. Scale bar = 400 µm.

Regarding the evidence that IL-6 signaling elicits the AnxA1/FPR1 axis leading in TNBC cells, we tested the in vivo effect of this receptor inhibition on tumor growth and fibroblast migration. The analysis of fibroblast migration under the influence of IL-6 present in supernatants of TNBC cells aims to find out the possible involvement of this cytokine in the establishment/remodeling of TME. Female nude mice bearing MDA-MB-231 tumors were treated with a control solution (PBS + DMSO 5%) or TCZ three times a week for one month while monitoring the primary tumor and metastasis. Figure 7A shows tumor growth over time. It was possible to observe that TCZ completely arrests tumor growth in a statistically significant manner (*p* < 0.001; Paired *t*-test). Figure 7B illustrates in vivo bioluminescence imaging of a tumor relapse and distal metastasis.

MDA-MB-231 and MDA-MB-157 cells were treated with a rIL-6, CsH, TCZ, and with STC, and the supernatants were used to treat HFF cells (Figure 7C). Fibroblasts migrated more rapidly in the presence of the supernatant from TNBC cells treated with rIL-6. The treatments with CsH, STC, and TCZ decreased migration properties when compared to their control cells. Indeed, CsH, as a FPR antagonist, inhibits AnxA1 signaling. Therefore, the inhibition of FPR1 and IL-6 modulated the motility of fibroblasts, and it can be considered an important factor for the TME. Even though CsH increases IL-6 that in turn increases secreted AnxA1, AnxA1 cannot signal through FPR1. Finally, the supernatants derived from the MDA-MB-231 cell line stably knocked down for AnxA1 (Figure S3) were used to evaluate the motility of HFF (Figure 7D). Quantifications of gap closures are reported in Figure S4.

## 4. Discussion

BC molecular classification is based primarily on the expression of ER, PR and HER2. This classification, in addition to improving diagnosis, drives targeted therapies such as hormonal therapy (e.g., Tamoxifen and Aromatase inhibitor) and HER2-targeted therapy (e.g., Trastuzumab) [43,44]. TNBC is the most aggressive BC subtype, displaying the worst prognosis and being responsible for a high incidence of metastases and low survival in patients with BC [45,46].

AnxA1 is a 37-kDa calcium-dependent phospholipid-linked protein that acts in the anti-inflammatory process and immune response. It is a downstream mediator of the actions of endogenous and exogenous glucocorticoids in cells. Many studies have shown that an increased expression of AnxA1 in TNBC and higher levels of AnxA1 have been observed in patients with familial BC, associated with the *BRCA1/2* mutation, poorly differentiated lesions, and with younger patients [47,48]. Furthermore, the AnxA1 expression was higher in lymph node metastases compared to primary tumors and correlates with BC aggressiveness. AnxA1 is expressed in the cytoplasm and nuclei and is secreted into the extracellular medium of TNBC cells, where it elicits an autocrine loop through its receptor, FPR1. Such autocrine signaling is essential in TNBC cell migration and invasion, thus, promoting metastases formation [16,23,49].

IL-6 is involved in immune regulation, inflammation, and oncogenesis. In previous studies, higher IL-6 levels were found to be important for TNBC tumor growth and metastases, and serum IL-6 levels increased with pathological grades. IL-6 binds to its receptor complex (IL-6R), inducing the dimerization of glycoprotein 130 (gp130) to promote the signal transducer and activation of STAT3 through the phosphorylation of Tyrosine 705 [50]. STAT3 is highly expressed and constitutively activated in TNBC cells, regulating the expression of their downstream target genes, promoting proliferation and tumor aggressiveness. IL-6/STAT3 signaling has been associated with tumor progression in BC and lung cancer by inducing EMT and angiogenesis [51]. Herein, we showed that the IL-6 expression was higher in TNBC samples and TNBC cell lines [52,53] and higher levels of IL-6 correlated with higher levels of AnxA1. In our work, besides confirming the existence of an autocrine signaling of AnxA1 in TNBC, we demonstrated that IL-6 activates this pathway. Indeed, we observed that the IL-6/STAT3 signaling pathway was responsible for inducing the secretion of AnxA1 and the subsequent activation of the AnxA1/FPR1 autocrine axis.

**Figure 8:**
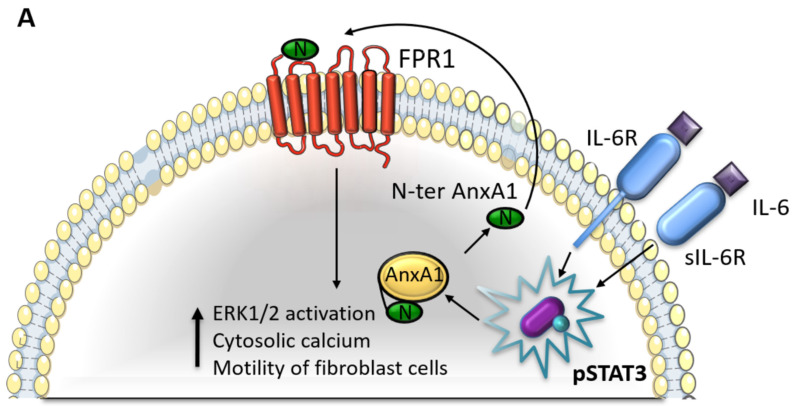

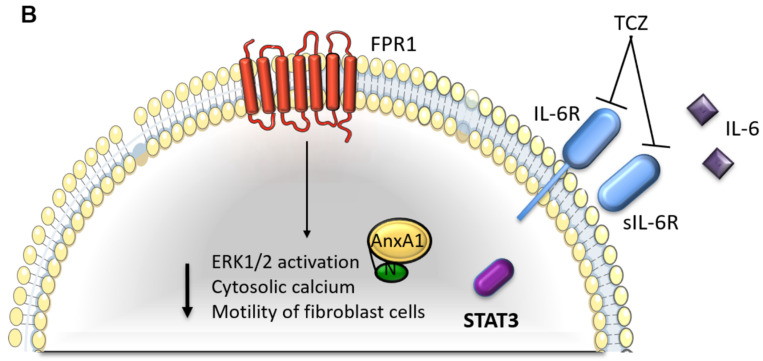
**Interaction between Annexin A1 (AnxA1) and Interleukin 6 (IL-6) in breast cancer.** (**A**) IL-6 signaling pathway in MDA-MB-231 and MDA-MB-157 cells. (**B**) Cellular events in MDA-MB-231 and MDA-MB-157 lineages after the inhibition of IL-6 signaling using Tocilizumab (TCZ), decreasing STAT3 phosphorylation levels (pSTAT3). FPR1: formyl peptide receptor 1; IL-6R: IL-6-receptor; sIL-6R: soluble form of IL-6 receptor.

According to previous studies, TNBC cells secrete autocrine IL-6 and are less responsive to paracrine IL-6 signaling, thus, these cells are less dependent on TME-derived IL-6. This exposure to IL-6 leads to the chronic induction of STAT3^pY705^, which promotes further growth and invasion of breast tumor cells [54,55]. TME is composed of signaling molecules from extracellular matrix elements (ECM), endothelial cells, cancer-associated fibroblasts (CAFs), and immune and inflammatory cells, which together play an important role in growth and invasion. Fibroblast cells in TME provide support for the migration, through the bloodstream, of cancer cells, contributing to metastasis [16,49]. TNBC is enriched with CAFs, which, in turn, contribute to immunosuppression [56,57]. In our study, we suggest that the interaction between the AnxA1 and IL-6 signaling pathways could be involved in the establishment of a tumor-promoting microenvironment. Indeed, when we treated HFF cells with the supernatants of AnxA1 or IL-6-inhibited TNBC cells, it resulted in a decreased migration. This result suggests that the IL-6 pathway could be involved in TME formation and the subsequent promotion of tumor progression due to its role in increasing the AnxA1 secretion. Secreted AnxA1, besides activating an autocrine loop in TNBC cells, could elicit signaling pathways in fibroblast, promoting the migration of this cell type. Probably due to this effect in promoting TME, we observed that TCZ suppressed TNBC growth in vivo. Our results are in line with Karakasheva et al., which demonstrated that TCZ suppresses tumor growth in vivo in part via the inhibition of STAT3 and MEK/ERK signaling, and IL-6 mediates the crosstalk between tumor cells and activated fibroblasts in TME [58]. In another study, IL-6 modulated the immune status of TME, increasing the antitumor effector function of CD8 + T cells and IL12 production by CD11c + dendritic cells, leading to increased metastatic colonization of colorectal cancer cells [59]. In the cancer context, it is known that the IL-6 is responsible to promote tissue invasion, EMT, acute-phase proteins and Th17 cells [60,61]. TCZ has a potent antiangiogenic effect and impedes TNBC cells to promote the differentiation of endothelial cells into network-like tubular structures in vitro, and impaired neovascularization in humanized breast orthotopic tumor xenografts. Therefore, TCZ could be a promising therapeutic target for TNBC [62]. In this study, we demonstrated and suggest that the AnxA1 and IL-6 pathways seem to act together in TNBC cells, leading to higher aggressiveness. The mechanism currently evaluated must be considered together with others that may interfere with the behavior of breast tumor cells; according to our results in Figure 8:

**(a) IL-6 pathway sustains the AnxA1 autocrine signaling in TNBC.** (1) IL-6 binds to its receptor (IL-6R) and stimulates STAT3 phosphorylation. (2) The IL-6/STAT3 signaling pathway stimulates the autocrine signaling of the AnxA1 N-terminal peptide (N-ter AnxA1) through FPR1. (3) Hence, AnxA1 induces a cytosolic calcium increase and ERK1/2 activation, thus, supporting BC aggressiveness. (4) Moreover, AnxA1 stimulates the motility of the surrounding fibroblasts, thus, contributing to the formation of a tumor-promoting TME.

**(b) The inhibition of the IL-6/STAT3 pathway decreases AnxA1 autocrine signaling and TNBC aggressiveness when IL-6 signaling is inhibited by TCZ.** (1) A decreased STAT3 phosphorylation is observed. (2) The lack of IL-6 signaling downregulates AnxA1 autocrine signaling, thus, leading to decreased ERK1/2 activation levels, decreased cytosolic calcium and aggressiveness and the inhibition of motility of the surrounding fibroblasts.

## 5. Conclusions

In summary, the present work proposes a functional interaction between AnxA1/FPR1 and IL-6 signaling pathways in TNBC. We demonstrated that in the MDA-MB-231 and MDA-MB-157 cells, the IL-6 signaling cascade modulates the activation of the AnxA1/FPR1 autocrine axis. We decided to use TCZ in our in vivo study as it is an FDA-approved drug, which reduced tumor growth and metastasis formation in nude mice bearing MDA-MB-231 tumors. We also described that the IL-6/AnxA1/FPR1 pathways influence TME by reducing fibroblast cell motility. In this sense, new experiments knocking down FPR1 and rIL-6 in TNBC cells can further confirm the important role of these receptors in cell survival and aggressiveness. Finally, IL-6 inhibition can be used as a possible therapeutic intervention and/or a new adjuvant therapy to improve the clinical outcome of patients with TNBC.

## Figures and Tables

**Table 1:** Comparison of expression levels of Annexin A1 (AnxA1) and Interleukin 6 (IL-6) according to clinicopathological parameters of breast cancer tissues. To calculate the Odds ratios, patients were divided into four subgroups by using the median of Iavg scores for AnxA1 and IL-6.

Characteristics	Patients			
	AnxA1 N (%)		IL-6 N (%)	
	High	Low	High	Low
**Age (year)**				
**≥50**	30 (28.3)	26 (24.5)	27 (25.5)	29 (27.4)
**<50**	18 (17)	32 (30.2)	26 (24.5)	24 (22.6)
***p*-value**	0.07		0.7	
**Odds Ratio**	2.1 95% CI (0.9–4.5)		0.8 95% CI (0.4–1.8)	
				
**ER**				
**Positive**	14 (13.2)	27 (25.5)	21 (19.8)	20 (18.9)
**Negative**	34 (32.1)	31 (29.2)	32 (30.2)	33 (31.1)
***p*-value**	0.07		0.8	
**Odds Ratio**	0.5 95% CI (0.2–1.1)		1.1 95% CI (0.5–2.4)	
				
**PR**				
**Positive**	10 (9.4)	18 (17)	14 (13.2)	14 (13.2)
**Negative**	38 (35.9)	40 (37.7)	39 (36.8)	39 (36.8)
***p*-value**	0.2		1.0	
**Odds Ratio**	0.6 95% CI (0.3–1.4)		1.0 95% CI (0.4–2.4)	
				
**HER2-enriched**				
**Positive**	18 (17)	21 (19.8)	15 (14.1)	24 (22.6)
**Negative**	30 (28.3)	37 (34.9)	38 (35.9)	29 (27.4)
***p*-value**	0.9		0.07	
**Odds Ratio**	1.1 95% CI (0.5–2.3)		0.5 95% CI (0.2–1.1)	
				
**Histological grading**				
**GIII**	5 (4.6)	8 (14)	6 (5.6)	7 (6.7)
**GI-GII**	43 (16.3)	50 (65.1)	47 (44.3)	46 (43.4)
***p*-value**	0.6		0.8	
**Odds Ratio**	0.7 95% CI (0.2–2.4)		0.8 95% CI (0.4–3.8)	
				
**Stage**				
**IIIA-IIIB**	11 (16.3)	19 (37.2)	16 (15.1)	14 (37.2)
**I-II (A-B)**	37 (4.6)	39 (41.9)	37 (34.9)	39 (36.8)
***p*-value**	0.3		0.7	
**Odds Ratio**	0.6 95% CI (0.3–1.4)		1.2 95% CI (0.3–2.7)	
**Tumor size (cT)**				
**T3-T4**	10 (9.3)	17 (20.9)	14 (13.2)	13 (12.2)
**T1-T2**	38 (11.7)	41 (58.1)	39 (39.6)	40 (37.7)
***p*-value**	0.3		0.8	
**Odds Ratio**	0.6 95% CI (0.3–1.6)		1.1 95% CI (0.5–2.6)	
				
**Lymph node (cN)**				
**N1-2**	33 (35.4)	36 (32.3)	27 (25.5)	42 (39.6)
**N0**	15 (16.7)	22 (15.6)	26 (24.5)	11 (10.4)
***p*-value**	0.5		0.003*	
**Odds Ratio**	1.3 95% CI (0.6 -3.0)		0.3 (95% CI (0.1–0.6)	
				
**Triple Negative**				
**Yes**	21 (19.8)	11 (10.4)	15 (14.1)	17 (16)
**No**	27 (25.5)	47 (44.3)	38 (35.9)	36 (34)
***p*-value**	* 0.007		0.7	
**Odds Ratio**	3.3 95% CI (1.4–7.9)		0.8 95% CI (0.4–2.0)	
				
**Luminal A**				
**Yes**	9	28	24 (22.6)	13 (12.2)
**No**	39 (36.8)	30 (28.3)	29 (27.4)	40 (37.7)
***p*-value**	* 0.002		* 0.03	
**Odds Ratio**	0.3 95% CI (0.1–0.6)		2.6 95% CI (1.1–5.8)	
				
**Luminal B**				
**Yes**	5 (4.6)	6 (5.7)	1 (0.9)	10 (9.4)
**No**	43 (40.6)	52 (49.1)	52 (49.1)	43 (40.6)
***p*-value**	0.9		* 0.02	
**Odds Ratio**	1.0 95% CI (0.3–3.5)		0.1 95% CI (0.01–0.7)	
				
**HER2 enriched**				
**Yes**	13 (12.2)	13 (12.2)	13 (12.2)	13 (12.2)
**No**	35 (33.1)	45 (42.5)	40 (37.7)	40 (37.7)
***p*-value**	0.6		1.0	
**Odds Ratio**	1.2 95% CI (0.5–3.1)		1.0 95% CI (0.4–2.4)	

ER: estrogen receptor; PR: progesterone receptor; HER2: human epidermal growth factor receptor 2; Iavg: average intensity of all pixels. * *p* < 0.05.

## Data Availability

Data are available within the article and its supplementary material.

## Supplementary Materials

The following are available online at https://www.mdpi.com/article/10.3390/cells11101705/s1: Figure S1: Immunohistochemistry staining negative control; Figure S2: Whole western blotting image staining; Figure S3: (A) Western blotting cropped images of AnxA1 expression in MDA-MB-231 expressing a shRNA control (shCRT) or a shRNA specific for AnxA1 (shAnxA1). (B) IL-6 expression levels in supernatants of MDA-MB-231 cells expressing shCRT or shAnxA1; Figure S4: (A) Quantification of gap closure of wound healing assay of HFF cells upon stimulation with supernatants of MDA-MB-231 cells treated as stated in the graph. (B) Quantification of gap closure of wound-healing assay of HFF cells upon stimulation with supernatants of MDA-MB-157 cells treated as stated in the graph. (C) Quantification of gap closure of wound-healing assay in MDA-MB-231 cells expressing a shRNA control (shCRT) or a shRNA specific for AnxA1 (shAnxA1). *p* < 0.05; * *p* < 0.001.
