# Supplementary material for: Interleukin-6 Signaling in Triple Negative Breast Cancer Cells Elicits the Annexin A1/Formyl Peptide Receptor 1 Axis and Affects the Tumor Microenvironment
Source: Cells. 2022 May 20;11(10):1705. doi: 10.3390/cells11101705 (PMC9139391; doi:10.3390/cells11101705)
Supplement: Supplementary file 1 [file cells-11-01705-s001.zip › cells-1445257-supplementary.pdf]

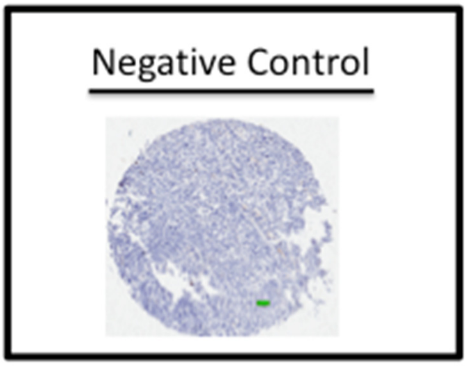

Figure S1. Immunohistochemistry staining negative control. Immunohistochemistry analysis of a TNBC sample incubated with all reagents but no primary antibody.

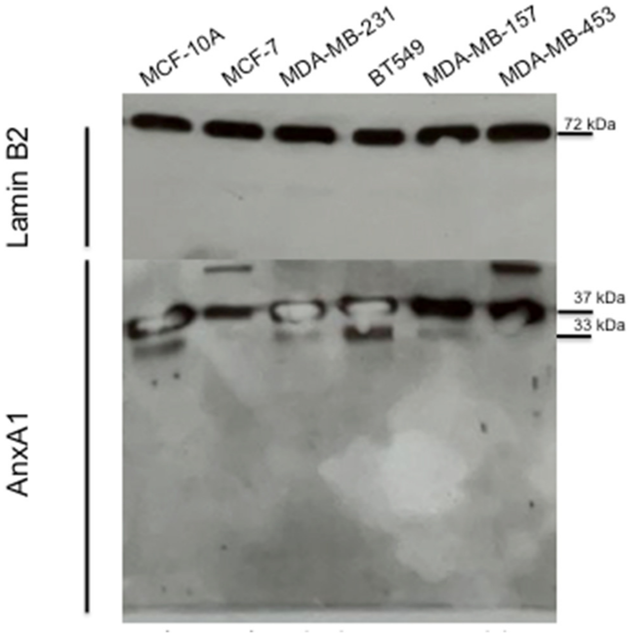

Figure S2. Whole western blotting image staining. AnxA1 and Lamin B2 staining in nuclear extracts of normal breast and BC cell lines. Membrane has been cut to incubate with anti-Lamin B2 or anti-AnxA1 antibody separately.

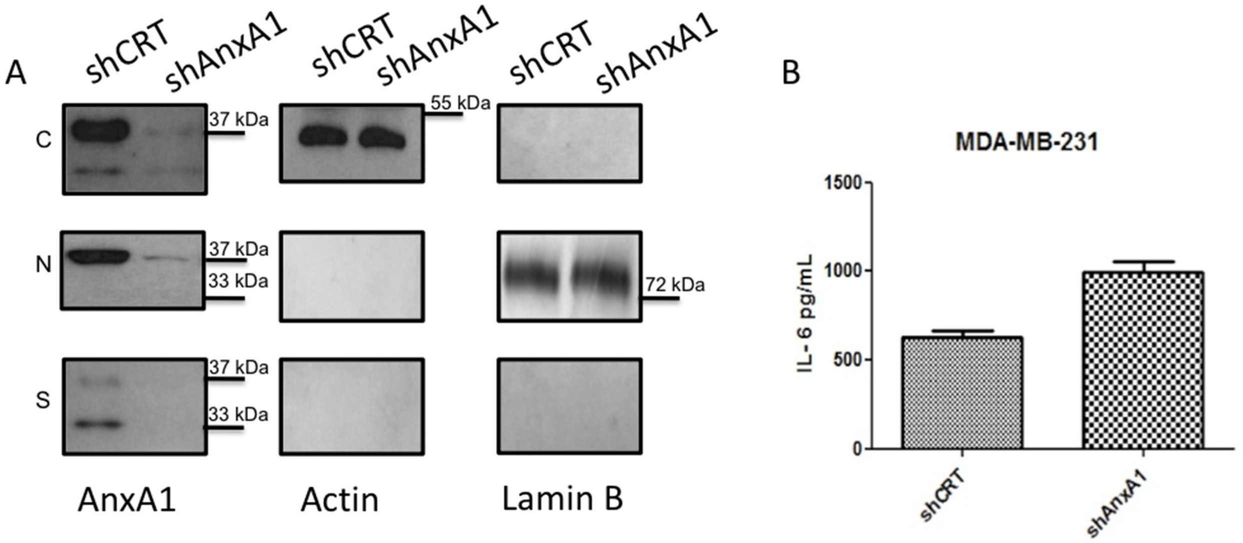

Figure S3. (**A**) Western blotting cropped images of AnxA1 expression in MDA-MB-231 expressing a shRNA control (shCRT) or a shRNA specific for AnxA1 (shAnxA1). (**B**) IL-6 expression levels in supernatants of MDA-MB-231 cells expressing shCRT or shAnxA1.

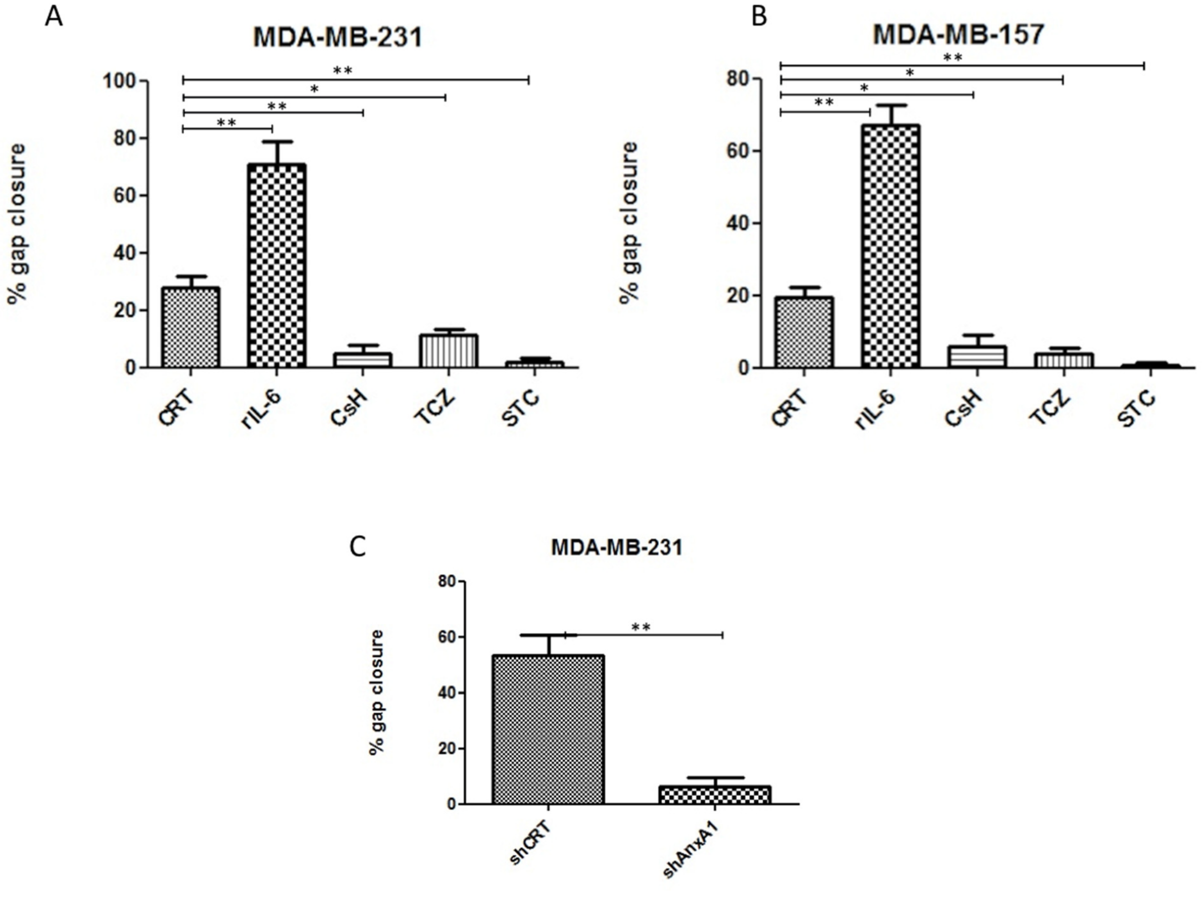

Figure S4. (**A**) Quantification of gap closure of wound healing assay of HFF cells upon stimulation with supernatants of MDA-MB-231 cells treated as stated in the graph. (**B**)

Quantification of gap closure of wound-healing assay of HFF cells upon stimulation with supernatants of MDA-MB-157 cells treated as stated in the graph. (**C**) Quantification of gap closure of wound-healing assay in MDA-MB-231 cells expressing a shRNA control (shCRT) or a shRNA specific for AnxA1 (shAnxA1). * $p < 0.05$; ** $p < 0.001$.
